# Catalogue of Medical Department of the University of Buffalo

**Published:** 1850-09

**Authors:** 


*Catalogue of Medical Department of the University of Buffalo.*—In the Catalogue of this Institution for 1849–50, recently issued, the name of a member of the Class was inadvertently omitted. The name referred to is J. S. Jamison, of Canisteo, Steuben Co., N. Y.

